# Endovascular Embolization of Sinonasal Tumors: A Report of Two Cases and a Technical Review

**DOI:** 10.7759/cureus.103339

**Published:** 2026-02-10

**Authors:** Toshiaki Furukawa, Hidetaka Onodera, Jun Isozaki, Katsumi Sakata, Tetsuya Yamamoto

**Affiliations:** 1 Department of Neurosurgery, Yokohama City University Hospital, Yokohama, JPN; 2 Department of Neurosurgery, Yokohama City University Medical Center, Yokohama, JPN; 3 Department of Radiation Oncology, Yokohama City University Hospital, Yokohama, JPN

**Keywords:** embosphere microspheres, endoscopic skull base surgery, preoperative embolization, sinonasal tumor, sphenopalatine artery

## Abstract

Hypervascular sinonasal tumors frequently cause recurrent epistaxis and pose a risk of significant intraoperative bleeding during resection. Preoperative embolization can improve operative visualization and safety; however, optimal technical strategies remain incompletely defined, particularly regarding embolic particle size and adjunctive flow-control techniques.

We describe a structured preoperative embolization approach for two patients with hypervascular sinonasal tumors. In both cases, superselective microcatheterization of sphenopalatine artery feeders was performed after confirming the absence of internal carotid artery (ICA) anastomoses. Embolization using 300-500 µm Embosphere particles (Merit Medical, South Jordan, UT, USA), combined with adjunctive proximal coil deployment, achieved complete devascularization. This protocol provided both distal tumor penetration and proximal flow control, serving, additionally, as an intraoperative landmark during endoscopic skull-base surgery.

These cases highlight a practical stepwise embolization strategy for sinonasal tumors, emphasizing careful angiographic assessment, appropriate particle sizing, and adjunctive coil placement. This technique supports safe and effective tumor devascularization and facilitates complete surgical resection in the endoscopic skull-base setting.

## Introduction

Sinonasal tumors represent a rare subset of head and neck neoplasms but are frequently hypervascular and associated with recurrent epistaxis and substantial intraoperative blood loss [1-4]. With the widespread adoption of endoscopic skull-base surgery, achieving reliable hemostasis and a clear operative field in this anatomically complex region has become increasingly critical for safe and complete tumor removal [5,6].

Preoperative endovascular embolization has emerged as a valuable adjunct to surgery for hypervascular sinonasal tumors, reducing intraoperative bleeding and improving surgical control [7-9]. However, the sinonasal arterial network exhibits extensive anastomoses between branches of the external carotid artery (ECA) and the internal carotid artery (ICA), particularly via the ethmoidal and ophthalmic arteries, creating a risk of inadvertent intracranial or ocular embolization [2,4]. While previous reports have emphasized the importance of superselective catheterization and careful angiographic assessment, specific technical approaches vary considerably among institutions [10,11].

Despite the increasing adoption of preoperative embolization, optimal strategies to maximize both procedural safety and completeness of surgical resection remain insufficiently defined. In particular, limited evidence is available regarding embolic particle size selection for sinonasal tumors, the role of adjunctive coil placement for proximal flow control, and standardized procedural endpoints tailored to endoscopic skull-base surgery. As endoscopic approaches continue to expand, clarifying these technical considerations has direct implications for clinical practice and perioperative outcomes.

In this report, we describe two cases of hypervascular sinonasal tumors treated with a structured preoperative embolization protocol incorporating superselective catheterization, 300-500 μm Embosphere particles (Merit Medical, South Jordan, UT, USA), and adjunctive coil placement. We highlight key technical nuances, rationale for embolic material selection, and perioperative results that support a stepwise embolization strategy for maximizing procedural safety and facilitating complete endoscopic tumor resection.

## Case presentation

Case 1. Esthesioneuroblastoma

**Figure 1:**
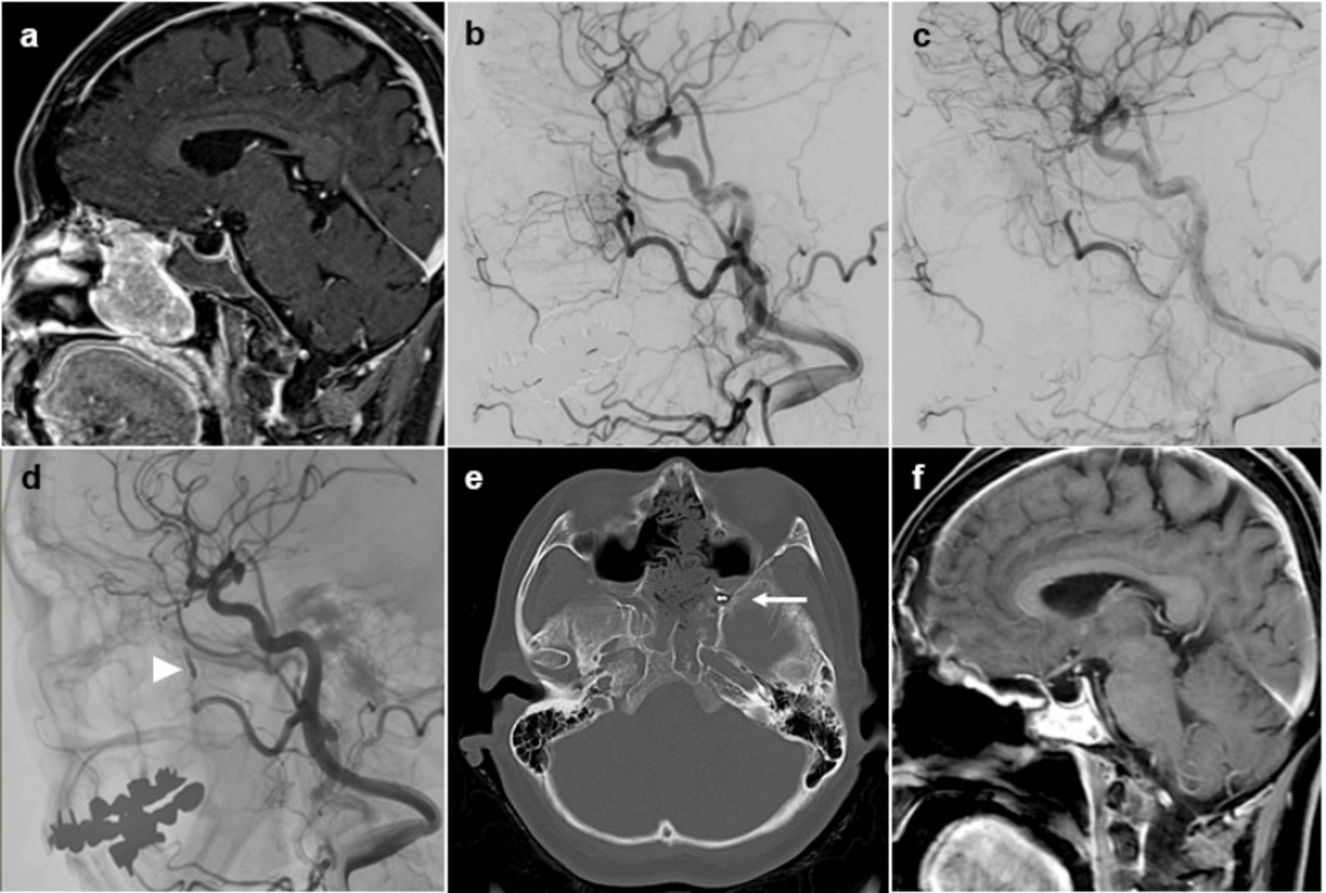
Pre- and postoperative multimodal imaging of Case 1 (esthesioneuroblastoma). (a-f) Preoperative MR image and angiography demonstrate hypervascularity of the tumor supplied by the sphenopalatine artery(a, b). Post-embolization angiography confirms disappearance of the tumor blush(c). Post-embolization and postoperative images show complete devascularization and coil placement at the feeding branch (arrowhead in d and arrow in e).

A 66-year-old woman with a medical history of bronchial asthma, meningioma, uterine myoma, ovarian cyst, and gastric ulcer presented with a one-year history of progressive nasal obstruction. Biopsy confirmed esthesioneuroblastoma. Imaging demonstrated a hypervascular mass centered in the left nasal cavity, with extension to the anterior skull base. Preoperative embolization was scheduled four days before resection (Figure 1).

Pre-embolization

Under local anesthesia, a 6-Fr RIST guiding catheter (Medtronic, Minneapolis, MN, USA) was introduced into the right ECA via a transradial approach. Angiography revealed a prominent tumor supply from the left sphenopalatine artery. An Excelsior SL-10 microcatheter (Stryker, Kalamazoo, MI, USA) was navigated to the distal portion of the feeding branch. Superselective angiography confirmed the absence of ICA anastomoses. Embolization was performed using 300-500 µm Embosphere particles (3.0 mL, diluted 1:4), followed by adjunctive coil deployment, resulting in complete elimination of tumor blush.

Surgery

Endoscopic resection was subsequently performed. Intraoperative bleeding was minimal, and gross-total resection was achieved, with excellent visualization.

Postoperative Course

The patient was discharged without complications or neurological deficits.

Case 2. Malignant sinonasal neuroendocrine carcinoma

**Figure 2:**
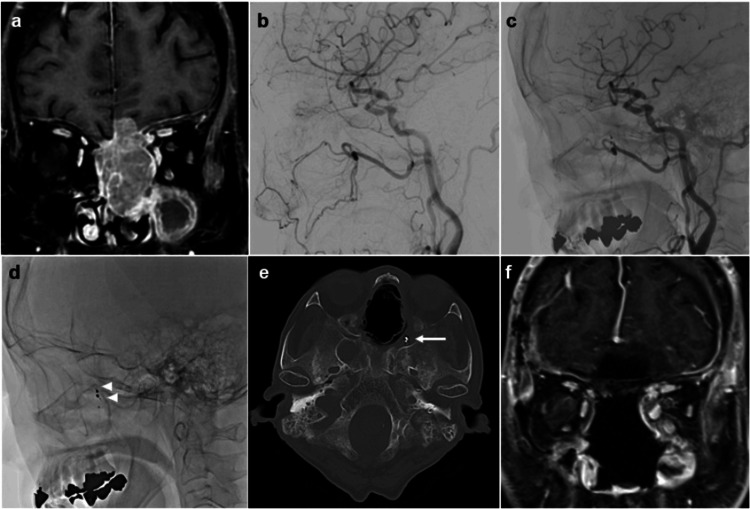
Pre- and postoperative multimodal imaging of Case 2 (malignant sinonasal neuroendocrine carcinoma). (a-f) Preoperative MR image and preoperative angiography demonstrate hypervascularity of the tumor supplied by the sphenopalatine artery (a, b). Post-embolization angiography confirms disappearance of the tumor blush and show complete devascularization (c and arrowhead in d). Postoperative CT bone image show coil placement at the feeding branch (arrow in e).

A 75-year-old woman with systemic lupus erythematosus and a history of bladder cancer presented with persistent epistaxis and a one-month history of anosmia. Nasal biopsy indicated sinonasal neuroendocrine carcinoma with intracranial extension, and combined endoscopic and transcranial resection was planned (Figure 2).

Pre-embolization

Under local anesthesia, a 4-Fr FUBUKI guiding sheath (Asahi Intec, Aichi, Japan) was advanced into the right ECA. Angiography demonstrated a prominent tumor blush arising from the left sphenopalatine artery. An Excelsior SL-10 microcatheter (Stryker) was positioned in the distal segment of the feeding artery. Superselective angiography confirmed no communication with the ICA. Embolization was performed with 300-500 µm Embosphere particles (4.0 mL, diluted 1:2), followed by coil placement, achieving complete tumor devascularization and occlusion of the feeding branch.

Surgery

The following day, a combined endoscopic and transcranial resection was performed. Preoperative embolization markedly enhanced visualization and minimized intraoperative bleeding, allowing complete tumor removal.

Postoperative Course

The patient recovered without new neurological deficits.

## Discussion

Preoperative embolization plays an essential role in the management of hypervascular sinonasal tumors by reducing intraoperative bleeding and facilitating safe and complete resection [5,6]. As endoscopic skull-base surgery continues to become increasingly prevalent, the establishment of technical principles tailored to the complex vascular anatomy and surgical challenges of the sinonasal region has gained clinical importance [7-9].

Accurate microcatheter positioning is a fundamental requirement for safe embolization of sinonasal tumors. Given the complex vascular anatomy of the sinonasal region, superselective angiography from the final catheter position is essential to delineate the dominant tumor feeders and to confirm the absence of branches supplying normal tissues or potentially dangerous anastomoses with ophthalmic or ICA branches. In both cases, this approach allowed clear identification of the sphenopalatine artery feeders. The sinonasal vasculature is known to harbor numerous potential collateral pathways - particularly via ethmoidal branches of the ophthalmic artery and ascending pharyngeal artery connections - and failure to recognize such microanastomoses may result in retinal ischemia or cerebral infarction [2-4].

The choice of embolic particle size represents another key technical consideration. We selected 300-500 µm Embosphere particles based on the typical caliber of branches supplying the sphenopalatine artery (approximately 200-800 µm) and the need to avoid distal migration into ophthalmic or intracranial collaterals [7]. Particles smaller than 200 µm may increase the risk of unintended intracranial penetration, whereas larger particles may result in predominantly proximal occlusion, with residual tumor perfusion [9]. In both cases, this particle size range achieved complete devascularization without ischemic complications, supporting its safety and effectiveness in this anatomical setting.

Adjunctive coil placement provided two distinct advantages. First, coils offered proximal vascular control, reducing the likelihood of delayed reperfusion and unexpected intraoperative bleeding. Second, coil deposition created a reliable endoscopic landmark, enabling surgeons to accurately identify the vascular pedicle deep within the surgical corridor. This benefit is particularly relevant in sinonasal tumors, where the limited working space of endoscopic skull-base surgery may restrict direct proximal vascular control. Collectively, these observations underscore the value of combining particulate embolization with targeted coil deployment in selected hypervascular skull-base tumors.

Although adjunctive coil placement was beneficial in our cases, its use should be guided by individual vascular anatomy and applied with caution in less commonly encountered situations, such as when feeders arise from very short or en passant branches supplying normal tissues, or when proximal occlusion could limit access to additional tumor feeders.

Collectively, these observations highlight three practical considerations for preoperative embolization of sinonasal tumors: (1) meticulous angiographic assessment for ECA-ICA collateral pathways, (2) accurate and stable superselective microcatheter positioning with real-time flow monitoring, and (3) selective use of adjunctive coil placement to provide both hemostatic support and surgical guidance. Together, these steps facilitated excellent visualization, minimal intraoperative blood loss, and complete tumor resection in both cases.

Although limited to two patients, this report demonstrates a reproducible, stepwise embolization workflow applicable to patients undergoing endoscopic resection of hypervascular sinonasal tumors. As embolization becomes increasingly integrated into multidisciplinary skull-base surgery, further accumulation of cases may help refine technical parameters and promote procedural standardization.

## Conclusions

Preoperative embolization using 300-500 µm Embosphere particles and adjunctive coil placement enabled safe and effective devascularization in two patients with hypervascular sinonasal tumors. A structured, stepwise approach - emphasizing superselective angiography, confirmation of potentially dangerous anastomoses, and a combined strategy of distal-penetrating and proximal-control embolization - facilitated complete endoscopic tumor resection with minimal blood loss. These cases support the value of a tailored embolization protocol designed to meet the anatomical and technical demands of sinonasal tumor surgery.
